# Effectiveness of tramadol or topic lidocaine compared to epidural or opioid analgesia on postoperative analgesia in laparoscopic colorectal tumor resection

**DOI:** 10.2478/raon-2025-0003

**Published:** 2025-01-04

**Authors:** Alenka Spindler-Vesel, Matej Jenko, Ajsa Repar, Iztok Potocnik, Jasmina Markovic-Bozic

**Affiliations:** 1 Clinical Department of Anaesthesiology and Intensive Care Medicine, University Medical Centre Ljubljana, Ljubljana, Slovenia; 2 Medical Faculty, University of Ljubljana, Ljubljana, Slovenia; 3 Department of Anaesthesiology and Intensive Care, Institute of Oncology Ljubljana, Ljubljana, Slovenia

**Keywords:** laparoscopic surgery, colorectal tumor, postoperative analgesia, topical analgesia, epidural analgesia, opioid analgesia

## Abstract

**Background:**

Chronic postoperative pain is the most common postoperative complication that impairs quality of life. Postoperative pain gradually develops into neuropathic pain. Multimodal analgesia targets multiple points in the pain pathway and influences the mechanisms of pain chronification.

**Patients and methods:**

We investigated whether a lidocaine patch at the wound site or an infusion of metamizole and tramadol can reduce opioid consumption during laparoscopic colorectal surgery and whether the results are comparable to those of epidural analgesia. Patients were randomly divided into four groups according to the type of postoperative analgesia. Group 1 consisted of 20 patients who received an infusion of piritramide. Group 2 consisted of 21 patients who received an infusion of metamizole and tramadol. Group 3 consisted of 20 patients who received patient-controlled epidural analgesia. Group 4 consisted of 22 patients who received piritramide together with a 5% lidocaine patch on the wound site. The occurrence of neuropathic pain was also investigated.

**Results:**

Piritramide consumption was significantly lowest in group 3 on the day of surgery and on the first and second day after surgery. Group 4 required significantly less piritramide than group 1 on the day of surgery and on the first and second day after surgery. The group with metamizole and tramadol required significantly less piritramide than groups 1 and 4 on the first and second day after surgery. On the day of surgery, this group required the highest amount of piritramide.

**Conclusions:**

Weak opioids such as tramadol in combination with non-opioids such as metamizole were as effective as epidural analgesia in terms of postoperative analgesia and opioid consumption. A lidocaine patch in combination with an infusion of piritramide have been able to reduce opioid consumption.

## Introduction

Chronic postoperative pain is one of the most common postoperative complications that severely impair patients’ quality of life. It occurs in about 10% of patients after major surgery and is a major health and economic problem. It typically starts as acute postoperative pain that is difficult to control and gradually turns into persistent neuropathic pain. Multimodal analgesics have the potential to reduce acute postoperative pain and target multiple points in the pain pathway. For this reason, postoperative pain management should be multimodal and opioid sparing.^1^ Thoracic epidural analgesia could alleviate pain after laparoscopic surgery.^2–4^ Although ERAS guidelines recommend the use of less invasive techniques for pain relief^5–8^, opioids are frequently used perioperatively despite their side effects.^9–11^ Non-opioids and 5% lidocaine patches applied topically could effectively reduce the use of opioids and their side effects.^12,13^

Indeed, efficient perioperative pain management is important to prevent late neuropathicain, even after laparoscopic lower abdominal surgery. The incidence is generally low compared to open surgery.^14^

In comparison to epidural or opioid analgesia, we wanted to investigate whether a lidocaine patch at the wound site or an infusion of metamizole and tramadol can reduce opioid consumption in laparoscopic colorectal surgery and whether the results are comparable to those of epidural analgesia. We also compared the incidence of postoperative neuropathic pain between the groups.

The primary outcome of this study was opioid consumption (piritramide) during the postoperative period, measured at three time points (immediately after surgery, the first postoperative day, and the second postoperative day). Secondary outcomes included pain assessment (VAS scores) and the incidence of postoperative neuropathic pain.

## Patients and methods

A prospective, randomised study with four parallel groups was conducted at the University Medical Centre (UMC) Ljubljana. The study included patients from the Clinical Department of Abdominal Surgery who were categorised as high-risk ASA (American Society of Anaesthesiologists) class 2–3 surgical patients. Adult patients who had undergone laparoscopic colorectal tumor resection were included in the study. Exclusion criteria included minors, pregnant women, patients undergoing laparotomy and patients undergoing palliative procedures.

The study was approved by the Slovenian National Medical Ethics Committee (151/03/09, 220/03/09, 148/06/11) and registered with Clinical Trials under the ID number NCT04719884.

Each patient was visited by a member of the research team one day prior to surgery to obtain informed consent and clarify any questions. Patients were randomised into four groups based on the type of postoperative analgesia. They were randomly assigned to one of four treatment groups using computer-generated random numbers. Randomization was performed prior to surgery by an independent statistician (simple randomisation was used), and allocation was concealed until the intervention was applied.

**FIGURE 1.**
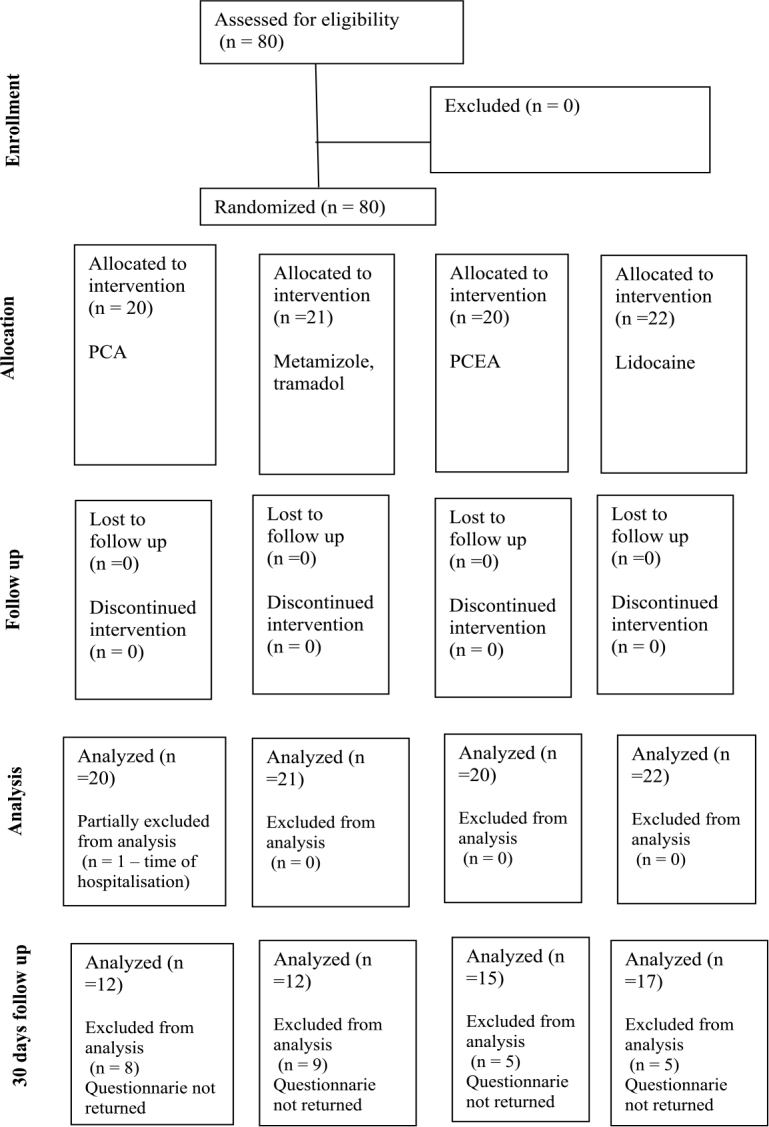
Consort chart of the study. Thet diagram shows the flow of participants through each stage of a randomized trial. PCA = patient-controlled analgesia; PCEA = patient-controlled epidural analgesia

Group 1 consisted of 20 patients who received an infusion of piritramide (patient-controlled analgesia, PCA). Group 2 consisted of 21 patients who received an infusion of metamizole and tramadol. Group 3 consisted of 20 patients who received patient-controlled epidural analgesia (PCEA). Group 4 consisted of 22 patients who received PCA together with a 5% lidocaine patch on the wound site (Figure 1).

Anaesthesia was performed by two anaesthetists, with the technique being uniform in all groups. Standard monitoring was performed. On admission, intravenous access was established, and patients were premedicated with midazolam. In group 3, a thoracic epidural catheter was inserted at the level of Th 7–8 in the left lateral position before the procedure and tested with 3 ml of 2% lidocaine.

Standard induction protocols were followed, including propofol (1–2 mg/kg) or etomidate (0.2 mg/kg), fentanyl (3–5 μg/kg) and vecuronium (0.1 mg/kg) or rocuronium (0.6 mg/kg). Anaesthesia was maintained with sevoflurane to keep the BIS value between 40 and 55. Analgesia was supplemented with fentanyl in groups 1, 2 and 4, while levobupivacaine 0.5% epidural was administered in group 2.

Muscle relaxation was monitored and vecuronium (2–4 mg) or rocuronium (10–20 mg) was administered depending on the TOF values. At the end of the procedure, the volatile agents were discontinued, and the muscle blockade was reversed with neostigmine (2.5 mg) and atropine (1 mg) or sugammadex (2 mg/kg).

Postoperative analgesia began during wound closure: in group 1 with PCA (piritramide 0.5 mg/ml; infusion 1.5 mg/h, bolus 1.5 mg, lockout 30 minutes), in group 2 with an infusion of tramadol 300 mg and metamizole 2.5 g (in 500 ml 0.9% NaCl, infusion rate 40 ml/h), in group 3 with PCEA (200 ml 0.125% levobupivacaine, 4 mg morphine, 0.075 mg clonidine; infusion 5 ml/h, bolus 5 ml, cut-off time 30 minutes) and in group 4 with PCA (piritramide 0.5 mg/ml; infusion 0.5 mg/h, bolus 1.5 mg, cut-off time 20 minutes) in combination with a 5% lidocaine patch on both sides of the wound. The plaster was removed after 12 hours and reapplied after a 12-hour break. In all groups, paracetamol 1g/6–8hrs iv was administered regularly. In groups 1, 3 and 4, metamizole 2,5g/12hrs iv was also prescribed. The prescribed analgesia in all four groups was not changed during the study, as it would have made it more difficult to evaluate the difference in additional bolus consumption of piritramide. We monitored the side effects of the analgesics. Appropriate antiemetic therapy was planned, but our patients did not require it. No significant sedative effects were observed.

After the operation, the patients were transferred to the post-operative care unit (PACU) and later to the intensive care unit of the abdominal surgery department. They received additional boluses of piritramide (3 mg) if required. The duration of the operation and the length of the wound were recorded intraoperatively. In the following two postoperative days, data such as visual analogue scale (VAS) scores, piritramide consumption, length of hospital stay and readmission to hospital were recorded. VAS was evaluated every six hours and when the additional piritramide bolus was needed.

The DN4 (*Douleur Neuropathique* 4) and Pain Detect questionnaires were used to assess the occurrence of neuropathic pain 30 days after surgery.

## Statistical analysis

The results were analysed with R: A language and environment for statistical computing. (R Foundation for Statistical Computing, Vienna, Austria). The ANOVA test was used to determine differences between the study groups. Pairwise comparisons were performed using the Dwass-Steel-Critchlow-Fligner test. A p-value of < 0.05 was considered statistically significant.

## Power analysis

A power analysis was performed to determine the appropriate sample size. Based on previous data from patients treated at our department and clinical relevance, we assumed a minimum effect size of 0.5 (Cohen’s d) for the reduction in opioid consumption between groups (based on previous data, this corresponds to 3mg of piritramide). This effect size was considered clinically significant. To detect this effect with 80% power and a significance level of 0.05, a total of 80 patients (approximately 20 per group) were required. The calculation was performed using standard formulas for comparing means in four independent groups (ANOVA).

## Results

We analysed the data of 20 patients in group 1, 21 patients in group 2, 20 patients in group 3 and 22 patients in group 4 (Figure 1). The general patient characteristics, length of wound and duration of surgery are shown in Table 1.

**TABLE 1.** General patients’ and procedure characteristics

	Group 1 (PCA)	Group 2 (tramadol-metamizole)	Group 3 (PCEA)	Group 4 (PCA and lidocaine)	^p^
Age (years)	59	65	60	59	0,394
Weight (kg)	76	75	79	76	0,833
Wound length (cm)	6,55	7,17	7,45	7,90	0,286
Duration of surgery (min)	139	133	117	112	0,024
Duration of hospitalization (days)	8	9	8	10	0,380
Day of first defecation	4	4	5	4	0,571

1 ANOVA test was used for comparison. p value of < 0.05 is statistically significant. PCA = patient-controlled analgesia; PCEA = patient-controlled epidural analgesia

The duration of surgery was significantly shorter in the lidocaine group (p = 0.024). There was no statistically significant difference between the characteristics listed in Table 1 with regard to the gender or ASA status of the patients. In each group, patients were equally distributed in terms of gender.

In group 1, there were 19 colon resections and 1 rectal resection. In group 2, there were 8 rectal resections and 12 colon resections. In group 3, there were 2 rectal resections and 18 colon resections, while in group 4, there were 3 rectal resections and 19 colon resections. All surgeries were laparoscopic. Patients in our study did not undergo additional anorectal excision during rectal surgeries. The duration of rectal surgeries and the length of postoperative wounds were comparable to bowel resections; therefore, we treated all surgeries as a group of laparoscopic colorectal resections.

There was no statistically significant difference in VAS scores between the groups. The VAS scores were low (below 3).

**FIGURE 2.**
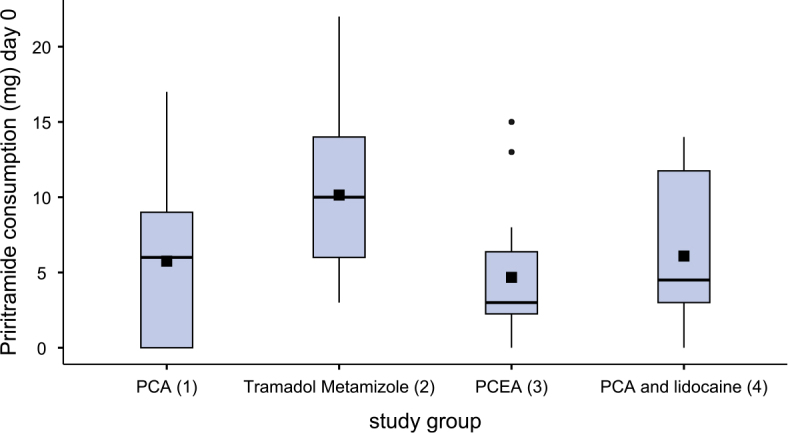
Piritramide consumption on day of surgery (day 0).

**FIGURE 3.**
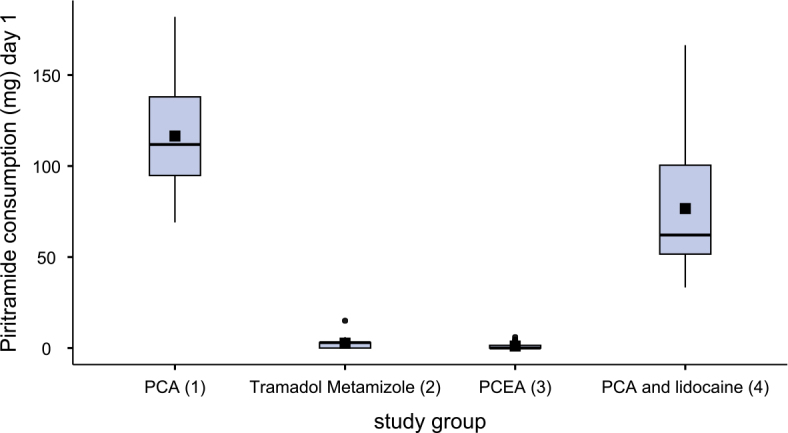
Piritramide consumption on first postoperative day (day 1). PCA = patient-controlled analgesia; PCEA = patient-controlled epidural analgesia

**FIGURE 4.**
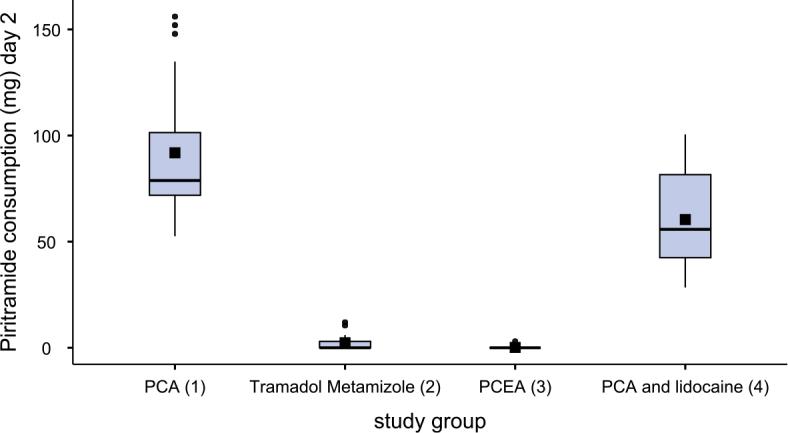
Piritramide consumption on second postoperative day (day 2). PCA = patient-controlled analgesia; PCEA = patient-controlled epidural analgesia

Figures 2–4 and Table 2 show the comparison of piritramide consumption on three consecutive postoperative days.

**TABLE 2.** Comparison of piritramide consumption between the group pairs

Comparison		P value (day 0)	P value (day 1)	P value (day 2)
PCA	PCEA	0.938	< 0.001	< 0.001
PCA	tramadol-metamizole	0.083	< 0.001	< 0.001
PCA	PCA + lidocaine	0.995	0.003	0.026
PCEA	tramadol-metamizole	0.008	0.352	0.038
PCEA	PCA + lidocaine	0.862	< 0.001	< 0.001
PCA + lidocaine	tramadol-metamizole	0.030	< 0.001	< 0.001

1 Dwass-Steel-Critchlow-Fligner pairwise comparisons. p value of < 0.05 is statistically significant. PCA = patient-controlled analgesia; PCEA = patient-controlled epidural analgesia

After surgery, patients in group 3 (PCEA) required less piritramide than patients in group 2 (tramadol-metamizole) (p < 0.08). There were no differences in piritramide consumption between patients in groups 2 and 3 on the first day after surgery, but on the second day after surgery, patients in group 3 required less piritramide than those in group 2 (p < 0.038). Similarly, patients in group 4 (PCA + lidocaine) required less piritramide than patients in group 2 (p < 0.03) on the day of the surgery. But on the first and second day after surgery, patients in groups 2 and 3 received statistically significantly less piritramide than patients in groups 1 and 4 (p < 0.001). Patients in group 4 required statistically significantly less piritramide than patients in group 1 both on the first day (p < 0.003) and on the second day after surgery (p < 0.026).

There were no significant differences between groups in Pain Detect or DN4 questionnaires scores using the Anova test (Table 3).

**TABLE 3.** The scores from Pain Detect and DN4 (*Douleur Neuropathique* 4) questionnaires in the study groups

Group (No. of answers)	Pain score (mean ± SD)	DN4 (mean ± SD)
PCA (12)	0	0
Tramadol and metamizole (12)	1.2 ±2.1	0.2 ± 0.4
PCEA (15)	0.1 ± 0.5	0.1 ± 0.4
Lidocaine (17)	0.06 ± 0.2	0

1 ANOVA test was used for comparison. p value of < 0.05 is statistically significant. DN4 = Douleur Neuropathique 4; PCA = patient-controlled analgesia; PCEA = patient-controlled epidural analgesia

## Discussion

Postoperative pain is managed in different ways in patients undergoing elective colorectal tumor resection, affecting patient outcomes and pain scores.

The epidural catheter provides superior analgesia for colorectal surgery, whether performed laparoscopically or with laparotomy.^15^ However, due to the frequent prolongation of the bowel recovery period and potential complications associated with catheter insertion, epidural analgesia is often replaced by other methods in minimally invasive procedures.^2,4,5,16^ Intravenous opioid-based patient-controlled analgesia (PCA) is a common method of postoperative analgesia, but peripheral analgesics could also be used to attenuate the side effects of opioids.^7,9,11^ Therefore, group 2 in our study received an infusion of the weak opioid tramadol and metamizole. We found that the consumption of piritramide was significantly reduced in this group on two consecutive days after surgery compared to group 1 (PCA) and group 4 (PCA + lidocaine). However, there was a significant requirement for additional opioids immediately after surgery. As expected, no additional analgesia was required in the epidural analgesia group.

Pain scores measured using the VAS scale were low (below 3), indicating adequate postoperative analgesia in all groups.

Several studies have shown that intravenous administration of lidocaine (for both laparoscopic and laparotomy procedures) improves postoperative analgesia in colorectal surgery, improves bowel function and shortens hospital stay.^6,17–22^ Studies have also shown potential benefits in terms of long-term cancer outcomes.^23^

Patients receiving lidocaine reported low pain scores, but piritramide consumption was relatively high due to the additional PCA infusion. It is likely that total opioid consumption would have been significantly lower if only PCA bolus infusions had been programmed.^22,24^

The use of lidocaine patches did not result in lower opioid consumption after thoracotomy and sternotomy.^25^ In a study of 103 patients undergoing elective laparoscopic colorectal surgery, thoracic epidural anaesthesia, spinal diamorphine and PCA were compared. It was found that the use of patient-controlled analgesia was associated with significantly higher postoperative pain scores and higher pain intensity.^26^

Recovery of bowel function after laparoscopic colorectal surgery was similar in the epidural analgesia and intravenous lidocaine groups, although epidural analgesia provided better pain relief.^27^

In our study, topical lidocaine was applied to the wound site in group 4. Compared to the PCA group, topical lidocaine also reduced piritramide consumption but had no favourable effects on bowel function, probably due to the local effect of lidocaine rather than systemic effects. No differences were observed in the postoperative recovery of bowel function in any of our groups. This finding is consistent with observations in another study of open colon resection, in which no differences were found between the epidural, intravenous opioid or intravenous lidocaine groups in terms of recovery of bowel function, length of hospital stay and postoperative pain control.^28^

67% of participants (56/83) completed pain questionnaires and no neuropathic pain was noted 30 days after surgery, which is consistent with observations from another study of laparoscopic colorectal surgery.^29^ The incidence of neuropathic pain is generally not expected in laparoscopic abdominal surgery and does not exceed 5%.^14^ However, the reported incidence of chronic postoperative pain after laparoscopic colorectal surgery is 17%, similar to laparotomy.^30^

## Conclusions

In laparoscopic colorectal tumor surgery, weak opioid tramadol in combination with non-opioid metamizole could be as effective as patient-controlled epidural analgesia (PCEA) in terms of postoperative analgesia and opioid consumption. A lidocaine patch in combination with an infusion of piritramide (PCA) could reduce opioid consumption.

## References

[1] Leslie JB, Viscusi ER, Pergolizzi JV, Panchal SJ. (2010). Anesthetic routines: the anesthesiologist’s role in GI recovery and postoperative ileus. Adv Prev Med.

[2] Pirie K, Traer E, Finniss D, Myles PS, Riedel B. (2022). Current approaches to acute postoperative pain management after major abdominal surgery: a narrative review and future directions. Br J Anaesth.

[3] Liu SS, Carpenter RL, Mackey DC. (1995). Effects of perioperative analgesic technique on rate of recovery after colon surgery. Anesthesiology.

[4] Novak-Jankovič V, Markovič-Božič J. (2019). Regional anaesthesia in thoracic and abdominal surgery. Acta Clin Croat.

[5] Reidel MA, Knaebel HP, Seiler CM, Knauer C, Motsch J, Victor N (2003). Postsurgical pain outcome of vertical and transverse abdominal incision: design of a randomized controlled equivalence trial [ISRCTN60734227]. BMC Surg.

[6] Herroeder S, Pecher S, Schonherr ME, Kaulitz G, Hahnenkamp K, Friess H (2007). Systemic lidocaine shortens length of hospital stay after colorectal surgery. Ann Surg.

[7] Kietzmann D, Bouillon T, Hamm C, Schwabe K, Schenk H, Gundert-Remy U (1997). Pharmacodynamic modelling of the analgesic effects of piritramide in postoperative patients. Acta Anaesthesiol Scand.

[8] Gustafsson UO, Scott MJ, Hubner M, Nygren J, Demartines N, Francis N (2019). Guidelines for perioperative care in elective colorectal surgery: Enhanced Recovery After Surgery (ERAS^®^) Society Recommendations: 2018. World J Surg.

[9] Salicath JH, Yeoh EC, Bennett MH. (2018). Epidural analgesia versus patient-controlled intravenous analgesia for pain following intra-abdominal surgery in adults. Cochrane Database Syst Rev.

[10] Lindberg M, Franklin O, Svensson J, Franklin KA. (2020). Postoperative pain after colorectal surgery. Int J Colorectal Dis.

[11] Angst MS, Clark JD. (2006). Opioid-induced hyperalgesia: a qualitative systematic review. Anesthesiology.

[12] Smoker J, Cohen A, Rasouli MR, Schwenk ES. (2019). Transdermal lidocaine for perioperative pain: a systematic review of the literature. Curr Pain Headache Rep.

[13] de Queiroz VKP, da Nóbrega Marinho AM, de Barros GAM. (2021). Analgesic effects of a 5% lidocaine patch after cesarean section: a randomized placebo-controlled double-blind clinical trial. J Clin Anesth.

[14] Shin JH, Howard FM. (2012). Abdominal wall nerve injury during laparoscopic gynecologic surgery: incidence, risk factors, and treatment outcomes. J Minim Invasive Gynecol.

[15] Perivoliotis K, Sarakatsianou C, Georgopoulou S, Tzovaras G, Baloyiannis I. (2019). Thoracic epidural analgesia (TEA) versus patient-controlled analgesia (PCA) in laparoscopic colectomy: a systematic review and meta-analysis. Int J Colorectal Dis.

[16] Guay J, Nishimori M, Kopp S. (2016). Epidural local anaesthetics versus opioid-based analgesic regimens for postoperative gastrointestinal paralysis, vomiting and pain after abdominal surgery. Cochrane Database Syst Rev.

[17] McCarthy GC, Megalla SA, Habib AS. (2010). Impact of intravenous lidocaine infusion on postoperative analgesia and recovery from surgery: a systematic review of randomized controlled trials. Drugs.

[18] Sun Y, Li T, Wang N, Yun Y, Gan TJ. (2012). Perioperative systemic lidocaine for postoperative analgesia and recovery after abdominal surgery: a meta-analysis of randomized controlled trials. Dis Colon Rectum.

[19] Harvey KP, Adair JD, Isho M, Robinson R. (2009). Can intravenous lidocaine decrease postsurgical ileus and shorten hospital stay in elective bowel surgery? A pilot study and literature review. Am J Surg.

[20] Kuo CP, Jao SW, Chen KM, Wong CS, Yeh CC, Sheen MJ (2006). Comparison of the effects of thoracic epidural analgesia and i.v. infusion with lidocaine on cytokine response, postoperative pain and bowel function in patients undergoing colonic surgery. Br J Anaesth.

[21] Paterson HM, Cotton S, Norrie J, Nimmo S, Foo I, Balfour A (2022). The ALLEGRO trial: a placebo controlled randomised trial of intravenous lidocaine in accelerating gastrointestinal recovery after colorectal surgery. Trials.

[22] Tikuišis R, Miliauskas P, Samalavičius NE, Žurauskas A, Samalavičius R, Zabulis V. (2014). Intravenous lidocaine for post-operative pain relief after hand-assisted laparoscopic colon surgery: a randomized, placebo-controlled clinical trial. Tech Coloproctol.

[23] Wall TP, Buggy DJ. (2021). Perioperative intravenous lidocaine and metastatic cancer recurrence-a narrative review. Front Oncol.

[24] Weibel S, Jelting Y, Pace NL, Helf A, Eberhart LH, Hahnenkamp K (2018). Continuous intravenous perioperative lidocaine infusion for postoperative pain and recovery in adults. Cochrane Database Syst Rev.

[25] Liu M, Wai M, Nunez J. (2019). Topical lidocaine patch for postthoracotomy and poststernotomy pain in cardiothoracic intensive care unit adult patients. Crit Care Nurse.

[26] Brown L, Gray M, Griffiths B, Jones M, Madhavan A, Naru K (2020). NoSTRA (Northern Surgical Trainees Reseach Association). A multicentre, prospective, observational cohort study of variation in practice in perioperative analgesia strategies in elective laparoscopic colorectal surgery (the LapCoGesic study). Ann R Coll Surg Engl.

[27] Wongyingsinn M, Baldini G, Charlebois P, Liberman S, Stein B, Carli F. (2011). Intravenous lidocaine versus thoracic epidural analgesia: a randomized controlled trial in patients undergoing laparoscopic colorectal surgery using an enhanced recovery program. Reg Anesth Pain Med.

[28] Swenson BR, Gottschalk A, Wells LT, Rowlingson JC, Thompson PW, Barclay M (2010). Intravenous lidocaine is as effective as epidural bupivacaine in reducing ileus duration, hospital stay, and pain after open colon resection: a randomized clinical trial. Reg Anesth Pain Med.

[29] Andjelkovic L, Novak-Jankovic V, Pozar-Lukanovic N, Bosnic Z, Spindler-Vesel A. (2018). Influence of dexmedetomidine and lidocaine on perioperative opioid consumption in laparoscopic intestine resection: a randomized controlled clinical trial. J Int Med Res.

[30] Joris JL, Georges MJ, Medjahed K, Ledoux D, lle Damilot G, Ramquet CC (2015). Prevalence, characteristics and risk factors of chronic postsurgical pain after laparoscopic colorectal surgery. Eur J Anaesthesiol.

